# Admission viscoelastic hemostatic assay parameters predict poor long-term intracerebral hemorrhage outcomes

**DOI:** 10.21203/rs.3.rs-4087284/v1

**Published:** 2024-03-29

**Authors:** Laura Sieh, Emma Peasley, Eric Mao, Amanda Mitchell, Gregory Heinonen, Shivani Ghoshal, Sachin Agarwal, Soojin Park, E. Sander Sander Connolly, Jan Claassen, Ernest E. Moore, Kirk Hansen, Eldad A Hod, Richard O. Francis, David Roh

**Affiliations:** Columbia University Vagelos College of Physicians and Surgeons; Columbia University Irving Medical Center; Columbia University; Columbia University Irving Medical Center; Columbia University Irving Medical Center; Columbia University Irving Medical Center; Columbia University Irving Medical Center; Columbia University Irving Medical Center; Columbia University Irving Medical Center; Columbia University Irving Medical Center; Denver Health Medical Center: Denver Health Main Campus; University of Colorado Denver; Columbia University Irving Medical Center; Columbia University Irving Medical Center; Columbia University Medical Center

**Keywords:** Intracerebral hemorrhage, viscoelastic hemostatic assays, Rotational Thromboelastometry, coagulation, outcome

## Abstract

**Background:**

Viscoelastic hemostatic assays (VHA) provide more comprehensive assessments of coagulation compared to conventional coagulation assays. While VHAs have enabled guided hemorrhage control therapies, improving clinical outcomes in life-threatening hemorrhage, the role of VHAs in intracerebral hemorrhage (ICH) is unclear. If VHAs can identify coagulation abnormalities relevant for ICH outcomes, this would support the need to investigate the role of VHAs in ICH treatment paradigms. Thus, we investigated whether VHA assessments of coagulation relate to long-term ICH outcomes.

**Methods:**

Spontaneous ICH patients enrolled into a single-center cohort study receiving admission Rotational Thromboelastometry (ROTEM) VHA testing between 2013 and 2020 were assessed. Patients with prior anticoagulant use or coagulopathy on conventional coagulation assays were excluded. Primary ROTEM exposure variables were coagulation kinetics and clot strength assessments. Poor long-term outcome was defined as modified Rankin Scale ≥ 4 at 6 months. Logistic regression analyses assessed associations of ROTEM parameters with clinical outcomes after adjusting for ICH severity and hemoglobin concentration.

**Results:**

Of 44 patients analyzed, mean age was 64, 57% were female, and the median ICH volume was 23 mL. Poor 6-month outcome was seen in 64%. In our multivariable regression models, slower, prolonged coagulation kinetics (adjusted OR for every second increase in clot formation time: 1.04, 95% CI: 1.00–1.09, p = 0.04) and weaker clot strength (adjusted OR for every millimeter increase of maximum clot firmness: 0.84, 95% CI: 0.71–0.99, p = 0.03) were separately associated with poor long-term outcomes.

**Conclusions:**

Slower, prolonged coagulation kinetics and weaker clot strength on admission VHA ROTEM testing, not attributable to anticoagulant use, were associated with poor long-term outcomes after ICH. Further work is needed to clarify the generalizability and the underlying mechanisms of these VHA findings to assess whether VHA guided treatments should be incorporated into ICH care.

## INTRODUCTION

Spontaneous intracerebral hemorrhage (ICH) carries the highest morbidity and mortality of all stroke subtypes^1,2^. These poor outcomes are largely driven by the volume of hemorrhage^3–7^. Thus, rapid correction of relevant coagulopathy is critical to prevent hematoma expansion (HE) and limit final ICH volume^8^. Current coagulopathy treatment paradigms in ICH rely on identifying preceding anticoagulant medication exposure, using plasma-based conventional coagulation assays (ie., PT: Prothrombin time, PTT: Partial Thromboplastin Time) to assess the “severity” of anticoagulation, which then guides hemorrhage control therapies. However, in most ICH patients not taking anticoagulants (~ 90%), conventional coagulation assays, including platelet counts, do not identify risk for HE or poor outcomes^9–11^. This highlights the known limitations of these tests in comprehensively assessing relevant coagulation systems and their interactions needed for blood clotting and hemostasis^12–14^. Such limitations for diagnosing coagulopathy relevant for HE and poor outcomes have prompted several trials testing various empiric hemorrhage control therapies in ICH. However, these interventions did not improve outcomes^15,16^ and even caused harm^17^. Given the risks of certain hemorrhage control therapies^18–20^, these findings emphasize a need to better diagnose coagulopathy relevant for ICH outcomes and thereby provide targeted and tailored treatment approaches^8^.

Unlike plasma-based conventional coagulation assays, which remove cells (platelets, red blood cells) to assess plasma coagulation protein contributions to clotting, viscoelastic hemostatic assays (VHAs) utilize a bedside, point-of-care platform to assess whole blood. Consequently, VHAs are able to assess the interactions between various cellular and plasma processes needed to initiate, develop, and stabilize blood clot. This provides a more global assessment of coagulation relevant for bleeding risk. In other critical illnesses and life-threatening hemorrhage, VHAs have identified coagulation processes relevant for bleeding risk and poor outcomes that are not seen using conventional coagulation assays^21–25^. Clinical VHA implementation in these settings allows for targeted, goal-directed hemorrhage control therapies, reduced unnecessary treatments, and improved clinical outcomes^26,27^. Thus, VHAs have become the standard of care in diagnosing coagulopathy in life-threatening perioperative and traumatic hemorrhage^28^. However, the role of VHAs in ICH management remains unclear and has been highlighted as a critical knowledge gap by the American Heart Association/American Stroke Association^8^. A better understanding of whether VHAs relate to clinical ICH outcomes is needed to clarify whether VHA-guided therapies can potentially improve these outcomes. Therefore, we sought to assess the relationships between VHA parameters and long-term neurological outcomes after ICH.

## METHODS

Consecutive ICH patients admitted between 2013 to 2020 to a single, large academic referral center were enrolled in a prospective ICH cohort study (ICHOP: ICH Outcomes Project). Baseline demographics, clinical characteristics, laboratory results, and outcomes were adjudicated in weekly multidisciplinary meetings of study physicians.

**Figure 1:**
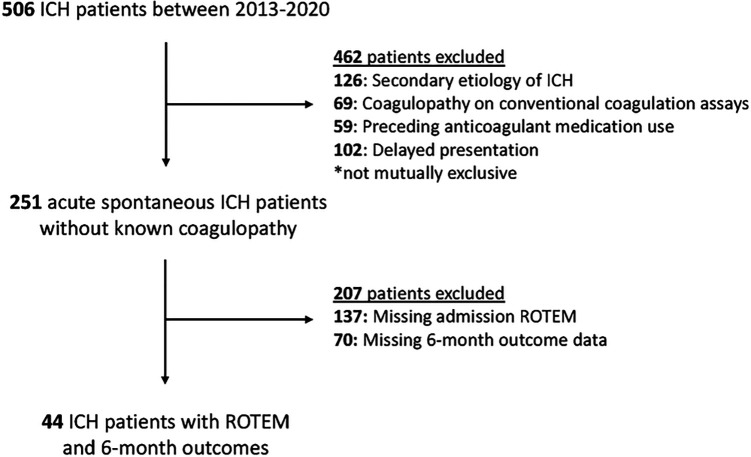
Patient selection and screening ICH: intracerebral hemorrhage; ROTEM: Rotational Thromboelastometry

Spontaneous ICH patients enrolled into this study with available admission VHA testing via the ROTEM delta device (Rotational Thromboelastometry, Instrumentation Laboratory, Bedford, MA) and 6-month outcomes were included for analyses. Patients with known or suspected secondary etiologies of ICH (vascular malformation, aneurysm, malignancy, ischemic stroke with hemorrhagic transformation), traumatic ICH, preceding anticoagulant use, coagulopathy identifiable using conventional coagulation assays (platelet count < 50 × 10^3^/μL, PT > 20 seconds, PTT > 50 seconds)^29–31^, and/or delayed presentation (> 24 hours after symptom onset) were excluded (Fig. 1). Patients were managed according to American Heart Association guidelines^8^ and center-specific treatment protocols as previously reported^31^.

### Rotational Thromboelastometry (ROTEM):

**Figure 2:**
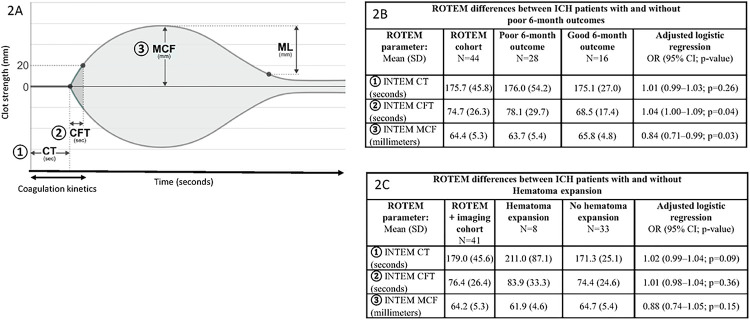
ROTEM parameters and results ROTEM: Rotational Thromboelastometry; SD: standard deviation; INTEM: intrinsic pathway assay; OR: odds ratio; CI: confidence interval **Figure 2A:** Primary ROTEM parameters assessed: (1) ROTEM Coagulation time (CT): time to initial fibrin clot formation from coagulation factor activation; (2) ROTEM Clot Formation Time (CFT): fibrin clot polymerization speed (from fibrin, fXIII, and platelets); (3) ROTEM Maximum Clot Firmness (MCF): clot polymerization/stabilization strength from platelets, fibrinogen, and fXIII. Additional ROTEM variables: Maximum Lysis (ML): early reduction of clot strength, fibrinolysis **Figure 2B:** Significant associations of slower coagulation kinetics (CFT) and weaker clot strength (MCF) with poor 6-month ICH outcomes. **Figure 2C:** Point estimates suggest associations of slower coagulation kinetics (CT) and weaker clot strength (MCF) with hematoma expansion.

ROTEM is an FDA-approved, point-of-care, VHA test of functional coagulation. In short, ROTEM detects developing fibrin clot over time by measuring impedance of a clotting whole-blood sample to rotational shear conditions from a rotating cylindrical pin. This allows ROTEM testing to provide a global assessment of plasma and cellular processes and their interactions needed to form a clot. ROTEM parameters assess: (1) coagulation kinetics (CT: coagulation time; CFT: clot formation time) from coagulation factor activation of fibrin clot initiation and fibrin polymerization, (2) clot polymerization strength (MCF: maximum clot firmness) from platelet, fibrinogen, and fXIII processes to stabilize and strengthen clotting, and (3) early clot lysis (ML: maximum lysis) from fibrinolytic vs. anti-fibrinolytic pathways (Fig. 2a). Separate assays evaluate these testing parameters specific to the intrinsic pathway (INTEM: contact activation), extrinsic pathway (EXTEM: tissue factor activation), and fibrinogen specific pathways (FIBTEM: cytochalasin-D platelet inhibition with tissue factor activation).

ROTEM was performed as part of admission coagulation testing, in addition to conventional coagulation assays, by trained personnel in the intensive care unit as a point-of-care device as previously specified^31^. Testing was performed on citrated blood within 60 minutes of collection according to the manufacturer’s instructions. Quality control, calibration, and operational checks were performed according to manufacturer policies. The primary exposure variables for our analyses were ROTEM assessments of coagulation kinetics (CT, CFT), and clot strength (MCF) in the INTEM assay given prior study findings^24,31^. Additional ROTEM parameters (i.e., ML) and assays (EXTEM, FIBTEM) were separately explored.

### Primary clinical outcome

Poor neurological outcome at 6-month, defined as modified Rankin scale (mRS) 4–6, was assessed as the clinical outcome. These outcomes were obtained by trained research coordinator teams using standardized telephone interviews.

### Radiographic hematoma expansion outcome

Hematoma volumes were measured semiautomatically using MIPAV software (NIH) on clinically obtained head computed tomographies^32^. Hematoma expansion (HE) was assessed as our secondary radiographic outcome and was defined as an increase in hematoma volume of ≥ 33% or ≥ 6 mL between baseline and final follow-up neuroimaging within 48 hours^7,32^.

### Statistical Analysis

Baseline characteristics of the study cohort were assessed with intergroup differences between poor and good 6-month outcomes analyzed using Fisher’s exact test or *χ*^2^-test for categorical variables and two-tailed t-test and Wilcoxon signed-ranked test for numerical variables. Multivariable logistic regressions assessed the relationships of ROTEM parameters with clinical 6-month outcome after adjusting for ICH score and baseline hemoglobin concentration, as we have previously identified that hemoglobin concentration separately associates with neurological outcomes^32^ and alters VHA tracings^33^. Sensitivity analyses were performed adjusting for antiplatelet medication use, baseline demographics, and ICH location. Additional analyses were performed to assess relationships of ROTEM with radiographic HE outcomes using similar regression models adjusting for baseline ICH volume. Statistical significance was judged at P < 0.05. Analyses were performed using SPSS.

## RESULTS

Of 44 ICH patients meeting criteria for analyses, the mean age was 64, 57% were female, 30% were white, and the median ICH volume was 23 mL. Table 1 describes intergroup differences between patients with and without poor 6-month outcomes. Patients with poor outcomes were notably older and had greater ICH severity. No other baseline characteristic differences were noted. We additionally assessed intergroup differences between our inclusion cohort and ICH patients excluded from analyses due to not receiving ROTEM to assess for any potential selection biases. We did not identify significant differences in baseline demographics, ICH severity, or long-term outcomes between patients receiving and not receiving admission ROTEM (supplemental table 1).

Intergroup differences in ROTEM parameters between patients with and without poor 6-month outcomes can be seen in Fig. 2b. In our adjusted regression models, we identified that slower, prolonged coagulation kinetics (adjusted OR for every second increase of CFT: 1.04, 95% CI: 1.00–1.09, p = 0.04) and weaker clot strength (adjusted OR for every mm increase in MCF: 0.84, 95% CI: 0.71–0.99, p = 0.03) in the intrinsic pathway assay (INTEM) separately associated with poor 6-month outcomes. Sensitivity analyses adjusting for pre-hospitalization antiplatelet medication, baseline demographics, and ICH location did not affect these associations (data not shown). Separately, when assessing other ROTEM assays of interest, we identified that slower, prolonged coagulation kinetics and weaker clot strength assessed in the extrinsic pathway assay (EXTEM) similarly associated with poor 6-month outcomes (supplemental table 2). No associations of fibrinolysis (ML) or fibrinogen strength (FIBTEM MCF) with 6-month outcomes were present.

In our secondary analyses, we identified 41 patients with complete neuroimaging data for HE assessments. In this sample, 20% of patients encountered HE. Though limited by a small sample size, patients encountering HE appeared to have slower ROTEM coagulation kinetics and weaker clot strength seen on intrinsic pathway assays (INTEM) compared to those not encountering HE (Fig. 2c). In our regression analyses, point estimates suggested that slower, prolonged coagulation kinetics, specifically coagulation time (adjusted OR for every second change in coagulation time: 1.02, 95%CI: 0.99–1.04, p = 0.09) and weaker clot strength (adjusted OR for every mm increase in MCF: 0.88, 95%CI: 0.74–1.05, p = 0.15) may be associated with HE in the intrinsic pathway assays, however these relationships were not statistically significant. When assessing other ROTEM parameters and assays, we did not identify an association of fibrinolysis (ML) with HE (supplemental table 3). However, we identified that greater fibrinogen assessments of clot strength were significantly associated with increased odds of HE (adjusted OR for every mm increase in FIBTEM MCF: 1.08; 95%CI: 1.01–1.17; p = 0.04).

## DISCUSSION

In our single-center cohort of spontaneous ICH patients, we identified novel findings that acute coagulation processes relevant for poor 6-month outcomes are identifiable on admission whole blood ROTEM VHA testing. Specifically, we identified slower, prolonged coagulation kinetics and weaker clot strength in patients who ultimately developed poor 6-month outcomes. These ROTEM VHA findings appeared to overlap in part with ICH patients encountering radiographic evidence of HE. These observations were independent to ICH severity and unrelated to anticoagulant medication use, suggesting the clinical utility of VHAs in diagnosing coagulopathy relevant for worse clinical and radiographic outcomes in ICH patients.

Our study excluded patients with evidence of coagulopathy on conventional coagulation assays or preceding anticoagulant medication use. And with these exclusions, we identified HE and poor 6-month outcomes in 20%, and 64%, respectively, mirroring estimates seen in larger multicenter studies^11^. Rather than suggesting that poor radiographic and clinical outcomes here were not related to potential coagulopathies, our ROTEM data highlights considerations that need to be made when assessing plasma-based conventional coagulation assays (PT/PTT). These plasma tests were originally designed to diagnose bleeding diathesis from specific coagulation factor deficiencies (i.e., hemophilia). Therefore, the PT and PTT assess in vitro activation of coagulation proteins within the canonical extrinsic and intrinsic pathways relevant for these diseases. While these tests are helpful in predicting bleeding risk under certain anticoagulant exposures where these pathways can be inhibited, they cannot predict bleeding risk from coagulopathies outside of these pathways (or from direct oral anticoagulants), and thus are not known to predict HE or poor outcomes after ICH. There are numerous other plasma protein processes, cellular systems (i.e., fXIII, von Willebrand Factor, functional fibrinogen, plasminogen activators, plasminogen activator inhibitors, platelet activity, red blood cells), and their relevant interactions which are not assessed using conventional coagulation assays, yet are critical for coagulation/hemostasis^12–14,34,35^. Many of these coagulation processes have been identified to be of relevance in ICH pathogenesis, HE, and outcomes using non-clinical, research based laboratory testing methods (i.e., genetics, enzyme-linked immunosorbent assays)^36–40^. In non-ICH settings, it is known these specific coagulation processes also impact VHA coagulation kinetics and clot strength^41–44^. This may suggest that whole blood VHAs can provide a more comprehensive clinical, bedside platform to identify these specific coagulation processes relevant for ICH outcomes, similar to what has been implemented in traumatic bleeding patients. However, further work will be required to comprehensively assess these specific coagulation proteins in conjunction with VHAs to be able to confirm specific coagulation processes and proteins responsible for relevant ICH outcomes that may become targetable in future approaches.

Though limited by sample size, there were notable overlapping observations of slower VHA coagulation kinetics and weaker clot strength in patients with both poor outcomes and increased HE. This could suggest that acute coagulation processes identifiable on admission VHAs relevant for long term outcomes may be driven (at least in part) by HE. And it could be posited that our specific findings suggest that adequate coagulation factor activation and clot strength from platelet polymerization are relevant processes needed to prevent HE and poor outcomes. While this work will require replication on a larger scale, it is notable that other ICH cohorts using alternative VHA testing modalities (i.e., Thromboelastography) have similarly identified relationships of impaired coagulation kinetics with HE^45^. In parallel, impaired platelet function, a contributor to VHA clot strength, has been shown to associate with both poor ICH outcomes and HE^46,47^.

However, it is important to note that VHA parameters relevant for both poor 6-month outcomes and acute HE in our study were not identically overlapping. It remains uncertain whether these VHA differences were merely due to our study’s small sample size or conversely due to true differences in coagulation processes separately relevant for acute radiographic and long-term clinical ICH outcomes. Given the dynamic changes of coagulation over time in ICH^39^, it is likely that acute coagulation processes, while relevant for acute HE, may not reflect the coagulation state changes that will occur temporally downstream and separately relate to other medical complications (i.e., thrombosis and infections) known to impact VHA tracings^48–50^ and long term ICH outcomes^9,51–53^. Furthermore, it should be considered that coagulation processes seen after ICH may be the result of the hemorrhage, rather than cause. Our data specifically showed that greater (not weaker) fibrinogen contribution to clot polymerization strength (FIBTEM MCF) was seen in patients encountering HE, but not in patients encountering poor outcomes. It could be speculated that acute hemostatic processes in patients with greater ongoing bleeding require greater activation of fibrinogen. With fibrinogen being a known acute phase reactant, it is possible that our elevated FIBTEM MCF reflects a reaction to these hemostatic demands rather than truly being a cause of ongoing bleeding as this would be counter to what would be expected clinically. And it remains to be determined whether fibrinogen “hyperactivation” in certain ICH patients results in a consumption of coagulation proteins and platelets leading to slower VHA coagulation kinetics, weaker clot strength, and in turn poor clinical outcomes, akin to what is seen in coagulopathies related to trauma or other critical illnesses^24^. Subsequently, further work will be needed to clarify coagulation changes over time in ICH as well as causal mechanisms underlying our VHA observations to be able to discern which coagulation proteins are separately and overlappingly relevant and targetable for HE and clinical ICH outcomes.

While our study strengths included the use of VHA ROTEM testing in a novel ICH cohort with 6-month outcome data and the exclusion of confounders like anticoagulant medication use, several limitations are worth mentioning. First and foremost, our single-center design and small cohort sample size was a limitation to the potential generalizability to our findings. While slower VHA coagulation kinetics and weaker clot strength have been related to poor outcomes in other non-ICH, critically-ill patients^24^, future work will be needed to replicate our VHA-clinical outcome relationships in a larger cohort of ICH patients. These limitations were similarly seen in our VHA-HE effect estimates. However, it was notable that our observations of slower VHA coagulation kinetics in patients with HE have been similarly described in other ICH cohorts^45^ attesting to the potential reproducibility/generalizability of our findings should a larger scale study be performed. Secondarily, our ROTEM VHA testing acquisition was based on a limited sample of convenience, which also raises questions about the generalizability of our data. Though we did not identify any clear intergroup differences between patients receiving or not receiving ROTEM over our study period to suggest a bias in how these tests were acquired, future prospective studies will need to be performed where rigorous VHA testing acquisition is performed across a larger ICH patient population. Third, our study was unable to address other potential measured or unmeasured confounders relevant to our observations. Though we eliminated patients with baseline coagulopathies attributable to anticoagulant use or medical disease presence, future studies will need to focus on serial VHA assessments and medical complications over the ICH hospitalization to capture dynamic changes of coagulation that may separately impact acute bleeding as well as long-term outcomes.

## CONCLUSION

We identified novel relationships between slower coagulation kinetics and weaker clotting strength on admission ROTEM VHA testing and poor long-term ICH outcomes. Further larger scale work is needed to characterize coagulation processes underlying these VHA observations and poor long-term ICH outcomes to assess whether these modalities can be used to guide coagulopathy therapies in ICH.

## Figures and Tables

**Table 1:** Baseline ICH characteristics of patients with poor vs favorable 6-month mRS

	All N = 44	6-month poor outcome N = 28	6-month favorable outcome N = 16	P-value
**Age**: mean (SD)	64 (14)	68 (12)	57 (13)	0.01
**Female:** N (%)	25 (57)	15 (54)	10 (63)	0.75
**Race/Ethnicity**: N (%)				0.26
White	13 (30)	9 (32)	4 (25)	
Black	13 (30)	5 (18)	8 (50)	
Asian	3 (7)	2 (7)	1 (6)	
Hispanic	9 (20)	7 (25)	2 (13)	
Other/Unknown	6 (14)	5 (18)	1 (6)	
**Medical history**: N (%)				
Dyslipidemia	13 (30)	10 (37)	3 (19)	0.31
Hypertension	36 (82)	21 (75)	15 (94)	0.22
Diabetes	9 (20)	6 (21)	3 (19)	1.00
Coronary artery disease	6 (14)	5 (19)	1 (6)	0.39
**Anti-platelet medication**: N (%)	18 (41)	13 (46)	5 (31)	0.36
**Clinical/radiographic**				
Baseline ICH Volume (mL): median (IQR)	23 (15–46)	28 (16–49)	18 (6–37)	0.14
IVH: N (%)	20 (45)	13 (46)	7 (44)	1.00
ICH Score: median (IQR)	2 (1–3)	3 (2–3)	1 (0–3)	0.01

ICH: intracerebral hemorrhage; mRS: Modified Rankin Scale (poor: 4–6; favorable: 0–3); SD: standard deviation; IQR: interquartile range; IVH: intraventricular hemorrhage.
